# Population density, a factor in the spread of COVID-19 in Algeria: statistic study

**DOI:** 10.1186/s42269-020-00393-x

**Published:** 2020-08-20

**Authors:** Nadjat Kadi, Mounia Khelfaoui

**Affiliations:** University of Khemis-Miliana, Khemis Miliana, Algeria

**Keywords:** COVID-19, Population density, Cluster analysis, Correlation, Simple regression analysis, Algeria

## Abstract

**Background:**

Since November 2019, the world has suffered the disastrous consequences of the COVID-19 pandemic. No country has been spared either socially or economically. Given the inevitability of the spread of this virus, researches have been active to understand and to counteract the factors that anticipate its spread. In this research, we endorse population density as a catalyst factor for the proliferation of COVID-19 in Algeria. We are interested in the relationship between population density and the spread of COVID-19 in Algerian cities. The latter is characterized by a disparity in the concentration of the population according to the geographic location of each.

**Results:**

The cluster analysis allowed us to isolate the groups of cities with the highest numbers of COVID-19 infected cases and the highest population densities. The regression models obtained suggest that there is a strong correlation between the population density and the number of COVID-19 infections in Algeria. This finding is verified by the correlation coefficients. Thus, it is estimated that population density has a positive effect on the spread of COVID-19 in the Algerian context during the study period.

**Conclusions:**

The spread of COVID-19 in Algeria is increasing as the population density increases. Once this factor has been demonstrated, the fight against the proliferation of the virus can be thwarted by measures to raise public awareness of social distancing in public places namely supermarkets, markets, and cafes.

## Background

Throughout history, humanity has encountered many epidemics that have forever transformed the social, economic, and architectural life of societies. The primary objective of these transformations is to curb the disease transmission within the population. Investigating the factors that cause this epidemic is the priority of the scientific community and epidemiologists considering the catastrophic human deaths and socio-economic effects on the population. Since November 2019, the world has been confronted to the COVID-19 epidemic commonly known as COVID-19. At the time of writing this article, COVID-19 caused 436,682 deaths and more than 8 million infected cases. The virus has spread to 213 countries according to the World Health Organization. A priori, people’s proximity is the first advanced factor to explain the transmissibility of pandemic viruses. Several research studies have been questioned on the link between the density of the territories and the current epidemic and which have concluded that the relation is positive in most of the time (Bouda-Olga 2020). As well, a study of European countries and the United States of America (USA) deduced that population density has a small but substantial effect on the rate of spread of the virus. She goes on to argue that there is a significant correlation of *R*^2^ = 0.23 in Europe and *R*^2^ = 0.39 in the USA (Babbit et al. 2020). Conversely, other analyses presume that the density of the population is not at issue and cannot be a determining factor in the proliferation of COVID-19. They present some cities like Singapore, Seoul, Shanghai, and New York as a counter-example because of their consequent population densities, and whose number of infections caused by COVID-19 is no different from that of cities with low urban density. Similarly, an empirical study in China on data collected for 284 Chinese cities, the results do not corroborate the idea that density is a key determinant of the risk of transmission of COVID-19. On either hand, the most afflicted cities are those with a relatively low density of between 5000 and 10,000 inhabitants per km^2^ (Fang and Wahba 2020).

Between supporter and opponent, the density of the population cannot be discounted in the research of the factors of propagation of the diseases and each country must focus on this element and all the related variables. For this, we propose research on the effect of population density on the spread of COVID-19 in Algeria. The choice of this factor is not fortuitous as long as the first instruction to avoid contamination by this virus is social distancing.

## Methods

- We calculate the population density for each city: it is the number of individuals or inhabitants occupying an area of 1 km^2^ (inhabitants/km^2^);
- To respond to the question of the study, we opted for cluster analysis and more precisely the hierarchical cluster method (Cornich 2007) in two cases:

1. To group the cities according to the COVID-19 infection rate in order to provide an idea of the spread of the disease and to make comparisons between the contamination rates of the different cities of Algeria;
2. Create clusters of cities according to the rate of COVID-19 infection and population density to allow comparisons between communities with the highest and lowest rates.

- We find the Pearson correlation coefficient (Schneider et al. 2010) and the coefficient of determination to determine the strength and the nature of the relationship between population density and the number of infected cases, through data for 48 cities, then according to geographic regions (coast, highlands, and the south);
- We construct a simple linear model (Schneider et al. 2010) to illustrate the impact of population density on the number of cases of COVID-19 infection over a period from 02 March 2020 to 10 June 2020. The linear model is as follows:

*Y* = *B* + a*X*

Such as:

- *Y* is the dependent variable that represents the number of cases of COVID-19 infection
- *X* is the independent variable that represents population density (inhabitants/km^2^)
- *B* is the constant

Then we deduce two models:

- Model 1: We use data from all cities to evaluate the relationship and degree of impact of population density on the spread of COVID-19 in Algeria;
- Model 2: In order to prove that population density is a factor in the spread of the virus in Algeria, we use data from cities that belong to the coastal region (which has 25 cities) whose population density is the highest (most of the Algerian population is concentrated on the coast).

We will use the IBM SPSS Statistics 25 to apply these statistical methods analysis.

### Data

Algeria is divided into 48 cities called Wilayas dispatched over an area of 2,381,741 km^2^ (World population prospects 2020), constituting three major regions: coastal cities, high plateau cities, and southern cities. The population density of each region is respectively 274 inhabitants/km^2^, 70.6 inhabitants/km^2^, and 2 inhabitants/km^2^ (National Statistics Office of Algeria (ONS) 2008). In addition, the coastal region contains 25 cities, the high plateau region is composed of 14, and the Sahara region consists of 9 cities (Ministry of Interior 2016).

Description of the various data included in the study:

- The population of each city: the data used are extracted from the last population census in Algeria for the year 2008 (National Statistics Office of Algeria (ONS) 2008);
- The area of each city: data from the interior ministry (Ministry of Interior 2016);
- Number of cases infected with the COVID-19 virus from the beginning of the epidemic in Algeria from 02 March 2020 to 10 June 2020 from the Ministry of Health, Population and Hospital Reform **(**Ministry of Health and hospital reform of Algeria 2020).

## Results

Until researching the relationship between population density and the spread of COVID-19 in Algeria, we propose to give an idea of the classification of cities by number of cases infected with the virus.

### Classification of cities according to cases of COVID-19 infection in Algeria

**Fig. 1:**
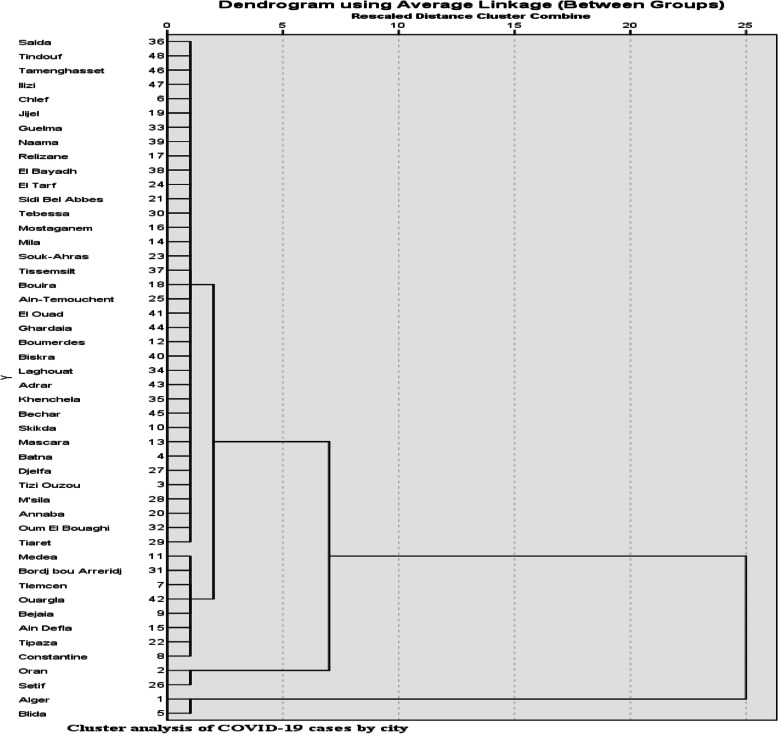
Cluster analysis of COVID-19 cases by city

The use of the classification method in data analysis has allowed us to classify cities according to the degree of cases infected with the virus in the study period. The results obtained are shown in Fig. 1.

Figure 1 shows that the study population of 48 cities was divided into four groups, namely:

- The first group: contains the 36 cities with an average of 105 infected cases;
- The second group: includes 8 cities (Constantine, Tipaza, Aindefla, Bejaia, Ouaragla, Telemcen, Bordj-Bouariridj, Medea), with an average of 332 infected cases;
- The third group: comprises the cities of Setif and Oran, with an average of 679 infected cases;
- The fourth group: includes the cities of Algiers and Blida, with an average of 1227 infected cases.

**Fig. 2:**
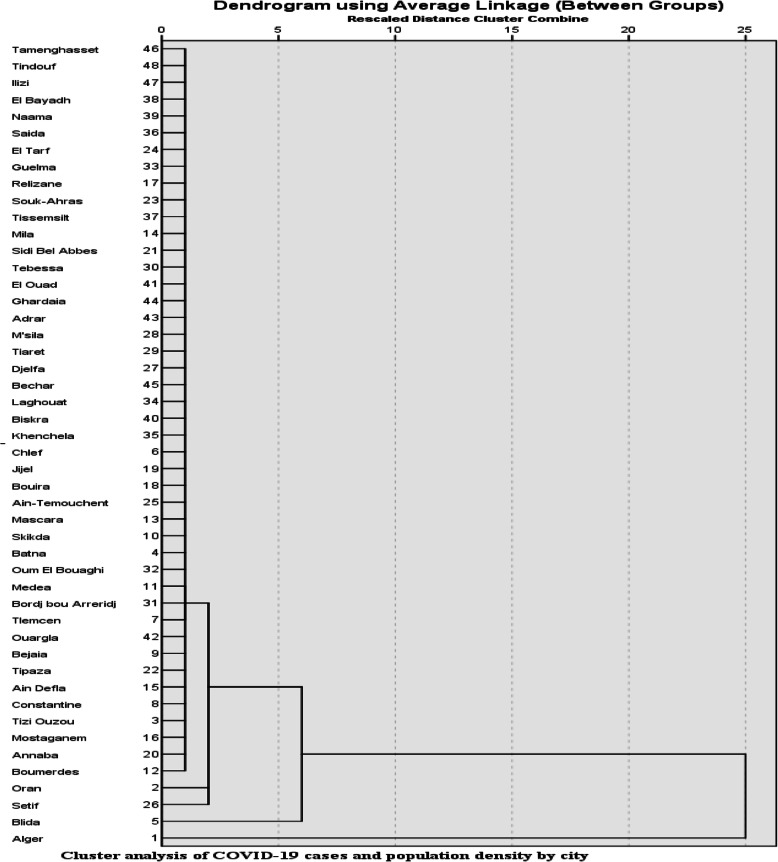
Cluster analysis of COVID-19 cases and population density by city

The cluster method enabled us to classify cities by cases of COVID-19 and population density for the study period. The results are illustrated in Fig. 2.

Figure 2 shows that the study population of 48 cities was divided into four groups, namely:

- First group: contains the 44 cities with an average population density estimated at 127.64 inhabitants/km^2^ and an average number of COVID-19 cases estimated at 146 cases;
- The second group: includes the cities of Oran and Constantine with an average population density of 407.325 (inhabitants/km2) and an average of 679 infected cases;
- The third group: comprises one city of Blida an estimated population density of 636.79 (inhabitants/km2) and 1288 cases of COVID-19;
- The fourth group: includes one city of Alger with an estimated population density of 2511.05 (inhabitants/km2) and 1166 cases of COVID-19.

### Correlation between population density and COVID-19 cases

The cluster method has allowed us to isolate the city groups characterized by a high population density number of COVID-19 cases and those with lower population density and cases of COVID-19.

So, can we say there is a relation between population density and COVID-19 spread?

Indeed, we note from Table 1 the existence of a strong relation between the two variables estimated at 0.711. The population density explains the spread of COVID-19 in Algeria at a rate of 50.50%. The rest of the contamination is explained by other factors: sanitary, social, and economic. It is also noted that this relation differs from one region to another. It is significantly strong in the coastal region (*R*^2^ = 0.531), marginally less strong in the high plateau region (*R*^2^ = 0.232), and relatively low in the southern region (*R*^2^ = 0.041).

**Table 1:** Correlation coefficients between population density and the COVID-19 cases

**Population density** **(inhabitants/km2)**	**Geographical regions**	**COVID-19 cases** **(Coronavirus)**	
		*R*	*R*^2^
	All the cities	0.711	0.505
	Coastal region	0.729	0.531
	Highland region	0.482	0.232
	Southern region	0.202	0.041

### The degree of influence of population density on the propagation of the COVID-19 in Algeria

**Fig. 3:**
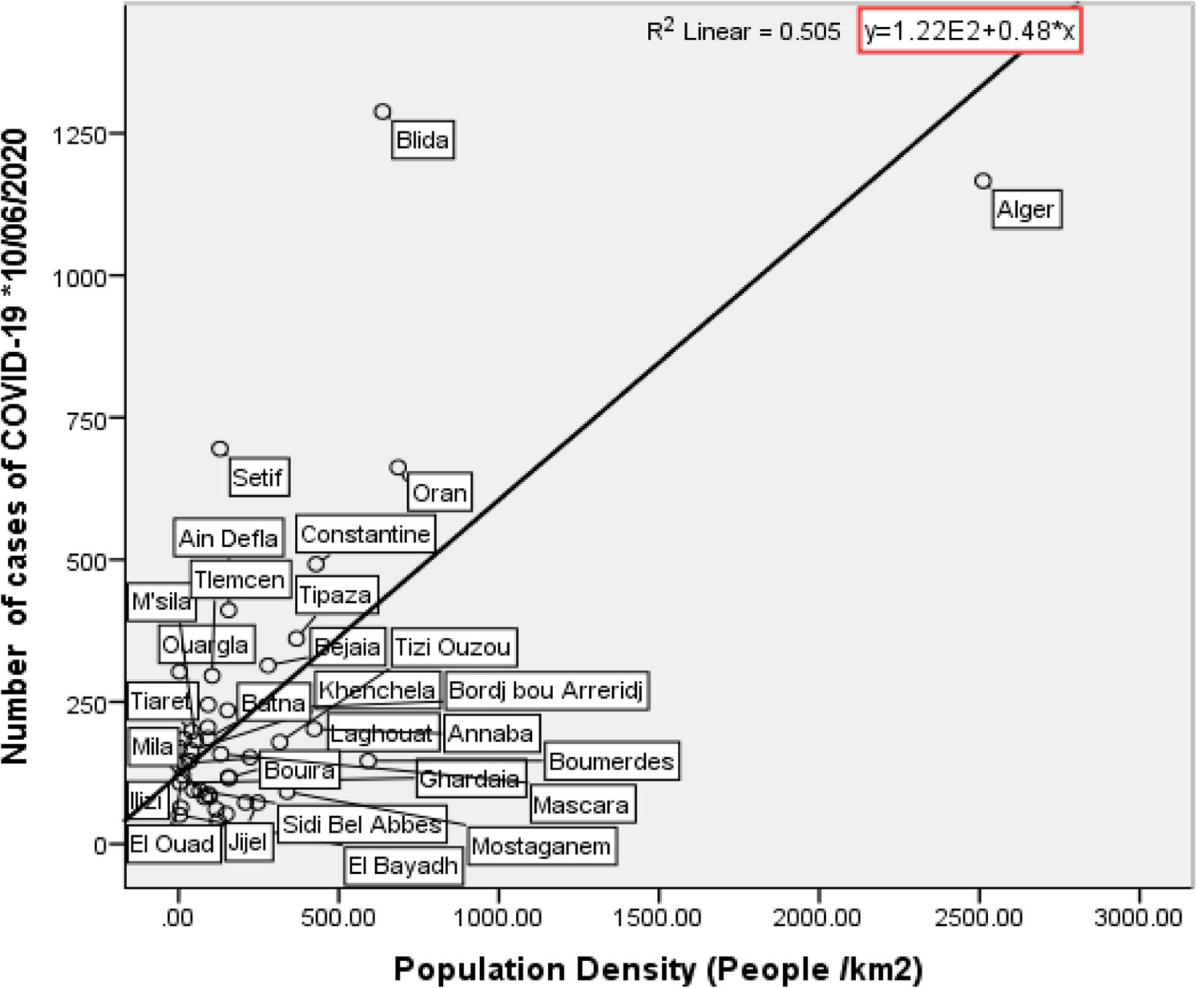
Representation of the point cloud of population density, COVID-19 cases, and the straight line of simple regression for all Algerian cities

**Fig. 4:**
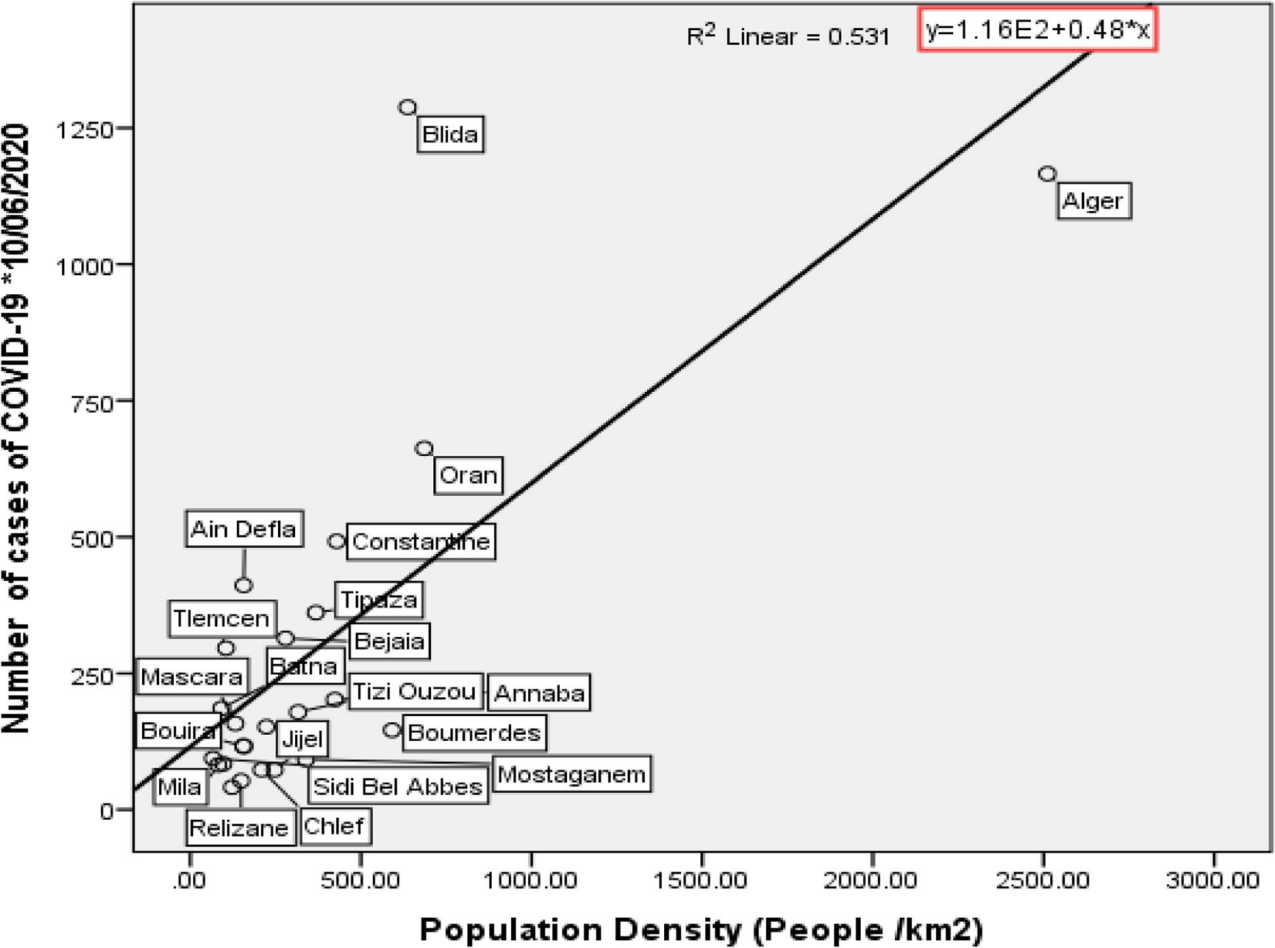
Representation of the point cloud of population density, COVID-19 cases, and the straight line of simple regression for coastal cities

In order to explain the degree of influence of population density on the spread of COVID-19 in Algeria, two clouds of points were created. The first concerns population density and cases of COVID-19 for all Algerian cities (Fig. 3). The second refers exclusively to coastal cities (Fig. 4).

Through Fig. 3, we note that is a significant effect at the significance level of 5% of the population density on the increase in infections with COVID-19 because each time the population density increases by 1 individual/km2, there is an increase in the number of cases of COVID-19 estimated at 0.48 for all Algerian cities (48 cities).

We note also through Fig. 4 that is a significant effect at the significance level of 5% of the population density on the increase in infections with COVID-19 because each time the population density increases by 1 individual/km2, there is an increase in the number of cases of COVID-19 estimated at 0.48 for coastal cities.

## Discussion

We note from the taxonomic analysis (Figs. 1 and 2) that the big Algerian cities (Algiers, Blida, Oran, and Setif) with a high population density are the most affected by the COVID-19 epidemic compared to the less populated cities. Also, the correlation coefficient indicates the existence of a strong correlation between population density and the number of cases of COVID-19 in the coastal region, compared with that of the highlands which is less important.

As for the southern region, the relationship between population density and COVID-19 spread is much lesser.

We found that population density has a positive effect on increasing the number of cases of COVID-19 virus. As population density increases, the number of infections increases, especially in coastal regions (Figs. 3 and 4).

## Conclusion

We deduce from this study that we carried out on the spread of the Covid-19 virus in Algeria over the period 02 March 2020 to 10 June 2020 that the higher the population density, the closer there is people in public places, which will increase the spread of the current virus.

Therefore, we recommend through this study to take into account the population density factor and to provide all necessary measures to protect against the spread of the virus in Algeria and highlight the cities that have a high population density. Also, increase awareness of social distance within the population realizing adequately that it is a very difficult task in our society; it is accustomed to the proximity inherited by ancestral traditions.

## Data Availability

The datasets used and/or analyzed during the current study are available from the corresponding author on reasonable request.

## Abbreviations

*R*: Correlation coefficient
*R*^2^: Coefficient of determination
